# The Role of CLEC-2 and Its Ligands in Thromboinflammation

**DOI:** 10.3389/fimmu.2021.688643

**Published:** 2021-06-09

**Authors:** Danyang Meng, Man Luo, Beibei Liu

**Affiliations:** ^1^ Department of Neurology, Affiliated Hospital of Jiaxing University, Jiaxing, China; ^2^ Department of Central Laboratory, Affiliated Hospital of Jiaxing University, Jiaxing, China

**Keywords:** CLEC-2, podoplanin, thrombosis, inflammation, thromboinflammation

## Abstract

C-type lectin-like receptor 2 (CLEC-2, also known as CLEC-1b) is expressed on platelets, Kupffer cells and other immune cells, and binds to various ligands including the mucin-like protein podoplanin (PDPN). The role of CLEC-2 in infection and immunity has become increasingly evident in recent years. CLEC-2 is involved in platelet activation, tumor cell metastasis, separation of blood/lymphatic vessels, and cerebrovascular patterning during embryonic development. In this review, we have discussed the role of CLEC-2 in thromboinflammation, and focused on the recent research.

## Introduction

CLEC-2 (CLEC-1b) is a type II transmembrane receptor (1, 2) of the C-type lectin superfamily, which are characterized by one or more C-type lectin-like domains (CTLDs). Members of the C-type lectin superfamily are mainly involved in growth and development, respiration, blood coagulation, angiogenesis and inflammation (3). CLEC-2 regulates multiple physiological pathways by recognizing and binding to both endogenous and exogenous ligands (4–6). While the role of CLEC-2 in tumorigenesis (7, 8) and platelet activation (9) is well-established, its involvement in thrombosis is ambiguous. Recent studies have implicated CLEC-2 in the inflammatory response, and correlated the upregulation of CLEC-2 ligands in the inflamed tissues with vascular integrity (10), which further highlights its role in thrombosis. CLEC-2 and its ligands are the molecular bridge between platelets, immune cells and target cells, and a novel mechanistic link between inflammation and thrombosis. Therefore, CLEC-2 related pathways are potential therapeutic targets for thromboinflammation.

## Molecular Structure and Cellular Distribution of CLEC-2

CLEC-2 is a platelet-activating type II transmembrane receptor with a molecular weight of ~32 kDa, and is highly expressed on megakaryocytes and platelets (1, 2). Originally known as snake venom protein receptor, CLEC-2 has a function similar to that of glycoprotein (GP) VI (GPVI) in activating Src (non-receptor tyrosine kinase) or Syk (spleen associated tyrosine kinase) upstream of phospholipase C (PLC) γ2 to trigger platelet aggregation (11–14). It comprises of a YXXL sequence, two conserved serine sequences at positions 21 and 27, and a partially conserved threonine sequence at position 9, of which YXXL is crucial for signal transduction (15). Binding of CLEC-2 to its cognate ligand triggers tyrosine phosphorylation of one intracytoplasmic YXXL motif, which activates the downstream semi-immunoreceptor tyrosine-based activation motif (ITAM) pathway (16). The semi-helical long loop region on the binding surface of CLEC-2 is variable compared with other parts, and binding of ligands can bring the cytoplasmic signal transduction domain of CLEC-2 closer to each other, thus promoting ligand induced dimerization (17). CLEC-2 binds to the tandem SH2 domain of Syk in a 2:1 stoichiometry based on its cytoplasmic tail phosphorylated peptide (18). Studies on transgenic mice have revealed that CLEC-2 is also expressed as relatively lower levels on Kupffer cells, sinusoidal endothelial cells (19), dendritic cells, macrophages (14), B lymphocytes and neutrophils induced during the inflammatory response (20). Lowe et al. demonstrated that the expression of CLEC-2 on neutrophils is likely the off-target effect of antibodies, and there are also statements the expression of CLEC-2 is probably limited to mice (20), whereas macrophages express CLEC-2 after phagocytosing platelets. Thus, the distribution of CLEC-2 has not been fully elucidated (5).

## CLEC-2 and Its Ligands

The major exogenous ligands of CLEC-2 are the snake venom toxin rhodocytin and type 1 human immunodeficiency virus (HIV-1). Rhodocytin is a heterodimeric C-type lectin that induces platelet aggregation through CLEC-2 clustering (14). In addition, the CLEC-2 expressed on platelets captures HIV-1 particles and leads to its subsequent phagocytosis, which is the basis of the high levels of circulating HIV-1 in infected individuals (21). CLEC-2 may interact with the cytokines within the HIV-1 rather than directly with the envelope protein (Env) of the virus. The direct interaction between CLEC-2 and HIV-1 was considered, however, CLEC-2 lacks a known amino acid motiv to regulate calcium complexation and carbohydrate binding of C-type lectins (1). Therefore, the structure recognized by CLEC-2 on the cell surface and on the HIV-1 particle was not revealed (21). There is evidence that the interaction between HIV-1 and CLEC-2 is indirectly mediated by an endogenous ligand (4). Recent studies have also confirmed that CLEC-2 does not recognize soluble HIV-1 Env, and the results show that virion incorporation of podoplanin was required for efficient CLEC-2-dependent HIV-1 interactions with cell lines and platelets. The binding of CLEC-2 to HIV-1 is indirectly accomplished through podoplanin, and it was also found that primary T cells may express a hitherto unrecognized ligand of CLEC-2, which is integrated into the viral Env and promotes HIV-1 transmission (4). Other exogenous ligands of CLEC-2 include the sulfated polysaccharide fucoidan (22) and diesel exhaust particles (23), although the mechanisms underlying their interaction remain to be elucidated.

**Figure 1:**
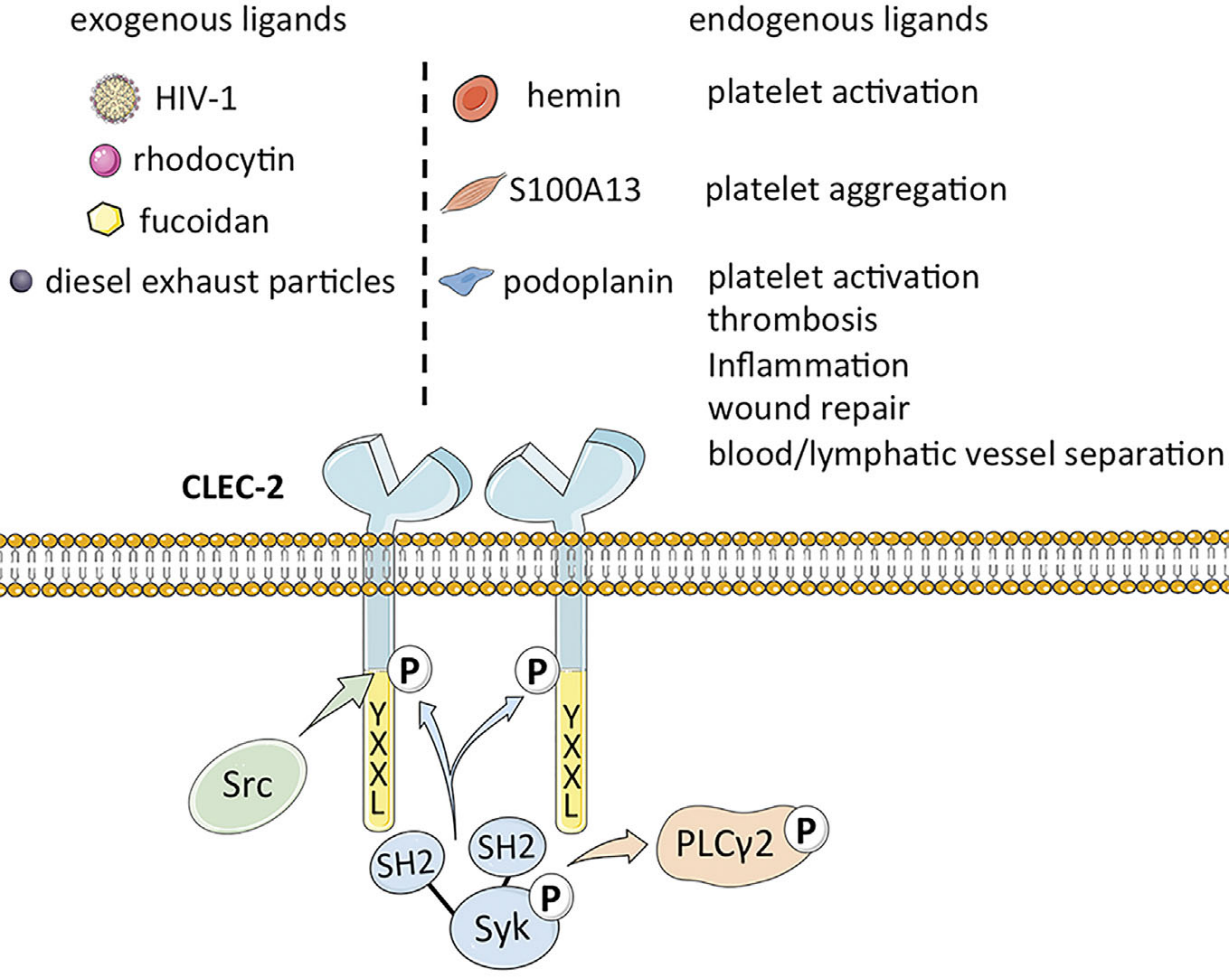
The structure of CLEC-2 and the function of its endogenous ligand.

Podoplanin (PDPN), also known as GP38 or Aggrus, is an endogenous ligand of CLEC-2 (24) that was first identified in rat glomerular epithelial cells (25). Podoplanin is a type I transmembrane glycoprotein (26) that mediates venous thrombosis (27), extravascular platelet activation and inflammation in atherosclerosis (27, 28), and wound repair (29) upon binding to CLEC-2. Furthermore, the CLEC‐2/podoplanin axis also facilitates blood/lymphatic vessel separation during embryonic development (30), maintains the lymph node vascular integrity, and optimizes adaptive immune responses (31). A recent study showed that the smooth muscle calcium binding protein S100A13 is a potential ligand of CLEC-2, and activates platelets independent of podoplanin (32). In addition, the hemin produced during the turnover of red blood cells (RBCs) can also activate platelets through integrin platelet glycoprotein IIb/IIIa receptor (GPIIb/IIIa) or CLEC-2 at low concentrations, and induce platelet aggregation at high concentrations, as well CLEC-2 can maybe promote pro-caogulant platelets which express phosphatidylserine (33). The ligands of CLEC-2 are summarized in **Figure 1**.

## CLEC-2 in Thrombosis

CLEC-2 is an important platelet activating receptor in the process of hemostasis and thrombosis (34–36). INU1, as an inhibitor of CLEC-2, can slightly prolong the tail bleeding time of mice, and CLEC-2 deficient platelets cannot form stable aggregates *in vitro* (34). In addition, the podoplanin/CLEC-2 axis regulates hemostasis and thrombosis (34–36) by promoting megakaryocyte proliferation and platelet formation (13, 19).

CLEC-2 and GPVI are two important receptors in platelet activation. HemITAM phosphorylation of CLEC-2 during the CLEC-2 process is mediated by the tyrosine kinase Syk, which is essential for signal transduction and downstream effector protein phosphorylation. Glycoprotein VI (GPVI), the center of platelet activation collagen receptor, has a pathway similar to CLEC-2, and its activation ultimately leads to platelet activation and thrombus growth. CLEC-2 may compensate for the lack of GPVI given that neither individuals with GPVI mutations nor the GPVI-knockout mice exhibit a hemorrhagic phenotype. Bender et al. showed that antibody-mediated blockade of either GPVI (JAQ1) or CLEC-2 (INU1) in mice down-regulated their expression and activity on the platelet surface, but did not affect bleeding, whereas simultaneous inactivation of both completely inhibited thrombus formation. The mutual compensatory action of the receptors indicates that targeted blocking of both can not only exert a strong anti-thrombotic effect but also impair normal hemostasis (36). In mice with suppressed Syk function, the increase in tail bleeding time was slight, reflecting the adhesion effect of CLEC-2 (37).

CLEC-2 plays a major role in process of thrombosis and a secondary role in normal hemostasis process. During wound healing, CLEC-2 may be useful in maintaining vascular integrity in the inflected skin only in the absence of GPVI (38). The ligand of CLEC-2 in the vascular system is not clear yet. Considering its undergo homophilic binding with submicromolar affinity, it is speculated that the endogenous ligand may be CLEC-2 itself (10, 35). It promotes the adhesion of platelets in vascular system, but in the case of inflammation, it plays a more important role in prevention of bleeding and thrombosis (10). There are corresponding changes in CLEC-2 in infectious and non-infectious diseases, especially in inflammatory reaction, CLEC-2 in platelets regulates the vascular integrity of acute inflammation (16), and its distribution varies with the anatomical location and inflammatory state of different immune cells (5), and there will be an up-regulation of CLEC-2 and its ligands (39, 40).

## CLEC-2 in Thromboinflammation

Thromboinflammation refers to the coordinated activation of thrombotic and inflammatory responses that manifests in various diseases, and is a major cause of mortality and morbidity. In the following sections, the specific roles of CLEC-2 and its ligands in neurological diseases, atherosclerosis, deep vein thrombosis, infectious thrombosis and cancer thrombosis have been discussed.

### CLEC-2 in Neurological Diseases

Platelets are central to the pathogenesis of ischemic stroke, which is characterized by a complex thromboinflammatory response triggered by the activation of platelets and immune cells, which destroys the blood-brain barrier (BBB) and leads to neuronal damage (41). GPVI plays an important role in the development of cerebral infarction by inducing an inflammatory response through the ITAM pathway (42, 43). Furthermore, the lectin-like oxidized low-density lipoprotein (LDL) receptor-1 (LOX-1) is elevated in the blood of patients with acute ischemic stroke and transient ischemic attack (44). The Syk cascade signal initiated by CLEC-2 is closely related to GPVI and belongs to C-type lectin receptor family with LOX-1. A study conducted on Chinese patients with acute ischemic stroke reported high levels of plasma CLEC-2, which was associated with poor prognosis and significantly increased risk of death (45). Other studies have also shown that plasma CLEC-2 is a predictor of cerebrovascular disease recurrence in patients with acute ischemic stroke (46). Podoplanin inhibitors mitigated the pathological changes after cerebral ischemia-reperfusion in a mouse model of middle cerebral artery occlusion-induced stroke, which indicates a potential role of the CLEC-2/podoplanin axis in thromboinflammation (47). In the same study, CLEC-2 and podoplanin were linked to the NLRP3 inflammasome (47). The above results were limited to cerebral artery embolization, and the role of CLEC-2 and its ligands in cerebral venous sinus thrombosis is still unclear.

Traumatic brain injury (TBI) is closely related to inflammation, hypercoagulation and apoptosis (48, 49). A significant proportion of patients with TBI have dysfunctional coagulation due to early platelet activation, leading to acute traumatic coagulation disease, which is systemic and affects many organs, arteries and veins (50–53). In addition, elevated plasma CLEC-2 levels in TBI patients correlate with poor prognosis, and can be used as a potential biomarker to evaluate disease severity and prognosis (54). However, in the mouse model, exogenous CLEC-2 appears to produce neuroprotective effects in TBI, improving brain edema, reversing blood-brain barrier damage and basement membrane degradation, and regulating inflammatory response, but does not improve the symptoms of neurological impairment in mice (55).

The underlying mechanisms of thromboinflammatory diseases induced by stroke and brain injury are not fully understood. GPVI and CLEC-2 expression in platelets is regulated by Src like adaptor (SLAP) and SLAP2, which are inactivated after cerebral ischemia, resulting in severe deterioration of neurological function after focal brain injury (56). Furthermore, the podoplanin/CLEC-2 axis has an endogenous role in the nervous system. Podoplanin is widely distributed in the central nervous system of mice from embryonic development to adulthood (57), and both podoplanin and CLEC-2 knockout mice show embryonic neurovascular development defects (9). In addition, podoplanin also plays an important role in neural progenitor cells proliferation and neuronal differentiation by interacting with the nerve growth factor (58). Podoplanin knockout mice show defects in cerebral neuron growth, synaptic plasticity and hippocampus-dependent learning and memory (59). Alzheimer disease (AD) patients have significantly higher levels of plasma CLEC-2 compared to those with mild cognitive impairment (MCI), which further underscores the role of GPIIb/IIIA-dependent platelet activation in cognitive diseases (60). Furthermore, podoplanin is overexpressed in the inflamed brain tissues of multiple sclerosis patients (61), indicating a specific role of podoplanin/CLEC-2 signaling in neuroinflammation. While a limited inflammatory response following brain injury promotes the repair of damaged tissues (62), unrestrained inflammation can lead to secondary damage and neuronal apoptosis (63). Podoplanin is up-regulated in the reactive astrocytes of mice with brain inflammation (64). Furthermore, rats injected with lipopolysaccharide (LPS) into their lateral ventricles exhibited neuroinflammation and upregulation of podoplanin in the neurons. Mechanistically, podoplanin promoted the re-entry of neurons into the cell cycle by altering the expression levels of cyclin D1 and cyclin-dependent kinase (CDK) 4, which triggered cell death and neuroinflammation (65). In addition to LPS, the pro-inflammatory cytokine TNF-α (Tumor necrosis factor-α) also upregulates podoplanin in the macrophages (66). However, it is unclear at present whether the podoplanin/CLEC-2-driven inflammatory changes following central nervous system injury are mediated through inflammasomes (NLRP3) or cytokines (IL-18, IL-1β, IL-6). Further studies are needed to explore the mechanisms underlying the thrombo-inflammatory responses induced by podoplanin and CLEC-2 using animal and cellular models.

### CLEC-2 in Atherosclerosis

Atherosclerosis refers to the gradual buildup of fats, cholesterol and other substances in the artery walls that eventually forms a plaque. It is not only associated with acute cardiovascular events but also involved in the pathological process of thrombosis after stent implantation. Studies increasingly show that the formation of atherosclerotic plaques is an inflammatory process that involves endothelial cell activation and adhesion of macrophages to the vascular wall, which eventually release large amounts of chemokines and cytokines. Multiple factors such as hypertension, lipid metabolism disorder and delayed wound healing can induce atherosclerotic build-up. In the late stages of atherosclerosis, the plaques rupture at the lesion and promote thrombosis, leading to cardiovascular events.

Thrombosis caused by atherosclerosis is related to platelet aggregation. Case-control trials have shown that elevated plasma sCLEC-2 is an independent risk factor for CHD (67, 68). Furthermore, podoplanin expression in smooth muscle cells and macrophages increases with atherosclerotic progression, and further aggravates the injury (69). The smooth muscle cells also express S100A13, which activate platelets through CLEC-2 independent of podoplanin (32). It has been suggested that S100A13 plays a more important role than podoplanin in early atherosclerotic lesions, given that podoplanin is rare in early atherosclerotic lesions (32). Injuries to vascular endothelium lead to S100A13 exposure, triggering platelet aggregation, and plaque induced ischemia and hypoxia, which in turn leads to inflammation. S100A13 is distributed on the surface of atherosclerosis, and podoplanin is detected in the interior of advanced atherosclerosis, and the different localization of S100A13 and podoplanin may be related to the different effects (69). S100A13 aggravates plaque growth from the outside.

The role of internal podoplanin is controversial. CLEC-2/PDPN-mediated phagocytosis is important for the formation of atherosclerotic plaques in mice, but the distribution may be species-specific, as noted in the same study (20). Podoplanin is present inside of the advanced atherosclerotic lesions rather than on the surface of the lesion, which blocks access to CLEC-2, and podoplanin may play an important role in triggering plaque rupture. In addition, podoplanin expressed on the stromal fibroblasts can also promote cell migration and invasion (70, 71), and induce inflammatory changes (72–74), indicating that the podoplanin/CLEC-2 axis is closely related to atherosclerotic progression (69). Local hypoxia and inflammation in the atherosclerotic plaque induces VEGF-A expression in smooth muscle cells (75), macrophages and endothelial cells (76), which enhances platelet aggregation and plaque erosion *via* the podoplanin/CLEC-2 axis (77). Furthermore, pro-inflammatory factors released from the intima of the arterial lumen into the blood activate CLEC-2 on the platelets and may trigger thrombosis (78). Patients with stent implantation are perpetually at risk of thrombosis and therefore require long-term dual anti-platelet therapy. Mice with CLEC-2 deficiency require a lower dose of antiplatelet drugs without an increase in the risk of bleeding (36), Therefore, blocking CLEC-2 can prevent stent thrombosis and slow down the progression of atherosclerosis. In addition, studies show that podoplanin is a marker of myocardial injury, and inhibition of podoplanin can accelerate recovery after myocardial infarction (74). Taken together, CLEC-2 and its ligands are promising therapeutic targets for cardiovascular diseases.

### CLEC-2 in Deep Venous Thrombosis

Deep venous thrombosis (DVT) is the formation of a blood clot in the deeper veins, usually in the lower extremities, following an aseptic inflammatory response (79) in the vascular endothelium. The endothelial cells in the intima of the blood vessels release Weibel-Palade bodies (WPB) containing cytokines, which mobilize platelets and white blood cells to the vessel wall, resulting in thrombosis (27, 80). The increase in blood pressure and turbulence caused by stenosis decreases the local blood flow, loosens the connection between vascular endothelial cells and increases vascular permeability. The resulting local hypoxia upregulates podoplanin in the vascular walls (81), which induces thrombosis through CLEC-2 (82). Thrombus formation is prevented in the absence of CLEC-2, which reduces platelet aggregation and white blood cell recruitment at the stenosis vessels. Podoplanin elevation after stenosis correlates with an increased risk of thrombosis, and further upregulation of podoplanin during thrombosis aggravates the condition. In addition, the weight and length of thrombus were significantly reduced in animals treated with the anti-podoplanin antibody (80). Up regulation of podoplanin and CLEC-2 is not only the cause of thrombosis, but also may be triggered by neutrophil-mediated inflammatory response. The reduced blood flow caused by stenosis and inducible factors produced by local hypoxia attract and activate neutrophils. In the early stage of DVT, multiple immune cells are recruited to the vessel wall, and platelets further enhance the inflammatory recruitment. Neutrophils recruited to the vascular wall release cytokines (83), further stabilize and promote the formation of thrombosis (27, 84). On the other hand, neutrophils also release metalloproteinases to further promote platelet aggregation and aggravate thrombosis (85). In this process, inflammation and thrombosis promote each other, leading to deep vein thrombosis (86).

Bruton’s tyrosine kinase (Btk) is an important non-receptor signaling kinase involved in platelet aggregation. Several Btk-dependent platelet aggregation pathways such as GPVI-activation by low collagen concentrations, FcgammaRIIA (an ITAM receptor) activation by cross-linking and VWF-stimulated GPIb activation are inhibited in human blood by low nanomolar IC50 concentrations of Btk-inhibitors (87). Besides, Btk is essential for platelet CLEC-2 (88). In heparin-induced thrombocytopenia, Btk inhibitors reduce CLEC-2- and GPIb-mediated platelet activation, monocyte interaction and activation, and neutrophil extracellular trap release (89). In addition, Btk inhibitors likely to reduce the microvascular and venous thrombosis in COVID-19 by blocking platelet CLEC-2, and well reduce thrombosis without an associated increase in bleeding (90), providing a new idea for the selective targeting of thrombotic inflammatory diseases.

### CLEC-2 in Septic Thrombosis

The role of podoplanin/CLEC-2 in inflammatory thrombosis was studied using mouse typhoid model by linking infection, inflammation and thrombosis (91). Podoplanin expressed on macrophages can activate the toll like receptor 4 (TLR4) signaling pathway *in vitro* (92, 93). Hitchcock et al. further showed that up-regulation of podoplanin in CLEC-2-dependent thrombosis is associated with TLR4/Interferon (IFN)-γ-dependent inflammation *in vivo*, and CLEC-2 is a key participant in this pathway (82). Bacteria in the liver cause a large number of macrophages depending on TLR4 and IFN - γ to aggregate, and ultimately damage the vascular endothelium due to the combined effects of bacteremia, infection, inflammation and cell migration. The platelets extravasating from the blood vessels are exposed to monocytes and other cells overexpressing podoplanin, and are activated *via* CLEC-2. Thus, inflammation triggered by bacterial infection directly induces thrombosis through the CLEC-2 pathway, which can be inhibited by knocking out CLEC-2 on the platelets or with anti-podoplanin antibodies.

A study on the septicemia mouse model showed that platelets limited the severity of symptoms through CLEC-2 signaling independent of thrombosis. The protective effect of CLEC-2 in septicemia was partly mediated by its interaction with the podoplanin expressed on inflammatory macrophages, which limited the infiltration of immune cells into the infected site by controlling cytokine/chemokine secretion, and mitigated organ damage. In addition, podoplanin reduces bacteremia by mediating the role of inflammatory macrophages. Podoplanin expressed in inflammatory macrophages not only regulates platelet aggregation, but also regulates the secretion of TNF-α in macrophages. Podoplanin antibody, mAb 8.1.1, regulates inflammatory response after sepsis. *In vitro*, mAb 8.1.1 reduced TNF-α secretion. However, *in vivo*, although pro-inflammatory cytokines and chemokines increased, the injection of anti-podoplanin antibody regulated the inflammatory response and immune cell infiltration during sepsis. Platelet CLEC-2 restricts the symptoms of sepsis by controlling multiple factors, including monocyte/macrophage migration, inflammatory mediators, bacteremia and organ damage, suggesting a complex role of platelets in regulating innate immunity against infection (94).

Some bacteria actively dissociate the thrombus to spread in the bloodstream, which suggests that inflammation-driven thrombosis is a novel strategy for bacterial capture and clearance (95). However, studies on animal models show that thrombosis controls the inflammatory diffusion in the early stage of infection (82, 94). During inflammation, platelet activation and thrombosis may adversely affect the host by inducing disseminated intravascular coagulation (DIC), and extensive microthrombosis occurs between arterioles and venules. Furthermore, if the thrombus exceeds a certain size, it may rupture and lead to other complications such as cerebral artery infarction or pulmonary artery embolism.

### CLEC-2 in Cancer Thrombosis

The interaction between CLEC-2 and podoplanin-expressing tumor cells promote angiogenesis, tumor growth and metastasis (96). Mice bearing lung tumors show extensive thrombosis, which can be alleviated by blocking CLEC-2. In addition, inhibition of CLEC-2 also reduced plasma cytokine levels, improved cachexia and prolonged survival of tumor-bearing mice (97). In the process of trying to deduce the mechanism of cancer-mediated inflammation, researchers found that podoplanin was up-regulated in the venous wall. Therefore, CLEC-2 may play an important role in tumor-induced thromboinflammation, and chronic long-term exposure to inflammatory cytokines induces podoplanin expression (98).

## Conclusion

The podoplanin/CLEC-2 axis promotes aseptic and bacterial inflammation, maintains vascular endothelial integrity and protects against microthrombosis. Although CLEC-2 is a potential target of anti-inflammatory drugs, targeting the CLEC-2 pathway may affect neovascularization during infection. Therefore, CLEC-2 blockade should only be considered as a short-term option. To better understand these diseases, relevant information is summarized in **Table 1**. Through the study of many diseases, it is obvious that it is very important to find the balance of CLEC-2 and its ligand in the treatment and application of thromboinflammation. Whether the related findings of CLEC-2 in these thromboinflammatory diseases can be widely applied to other diseases, and whether the involved pathways can specifically target to improve the status of thromboinflammatory diseases are still the focus of our consideration and research. However, the important effects of CLEC-2 and its ligands on thrombosis and inflammation cannot be ignored, and the research on the mechanism of CLEC-2 and related drugs will still be the future research hotspot in this field.

**Table 1:** CLEC-2 in thromboinflammation.

Diseases	Species	Trend	Outcomes	Potential molecules	References
Ischemic stroke	Human	↑	High risk of death Poor prognosis	SLAP, SLAP2, podoplanin, IL-1β, IL-18, NLRP3, TNF-α	(45, 46)
	Mouse	↑	Regulate inflammatory cytokines Poor prognosis		(47, 56, 66)
Traumatic brain injury	Human	↑	Serious illness Poor prognosis		(54)
	Mouse	↑	Neuroprotection Cerebral edema improved Regulation of inflammatory response		(55)
Atherosclerosis	Human	↑	High risk of coronary heart disease Stent thrombosis	Podoplanin, VEGF-A, S100A13	(32, 67, 77)
	Mouse	↑			
Deep venous thrombosis	Mouse	↑	Severity of thrombosis	Podoplanin	(86)
Sepsis	Mouse	↑	Limit disease severity Limit organ damage Reduce bacteria	Podoplanin, TLR4, IFN-γ	(82, 91, 94)
Tumor	Human	↑	Promote tumor angiogenesis, growth and metastasis	Podoplanin, TNF-α	(96, 97)

## Author Contributions

DM drafted and edited the manuscript. ML and BL edited the manuscript. All authors contributed to the article and approved the submitted version.

## Funding

This work was supported by the Key Supporting Discipline-Neurology (2019-fc-04) in Jiaxing, Jiaxing Public Welfare Research Program (2021AY30022), and the “Morning Star” project (2020-QMX-23) of the Affiliated Hospital of Jiaxing University.

## Conflict of Interest

The authors declare that the research was conducted in the absence of any commercial or financial relationships that could be construed as a potential conflict of interest.
